# Dynamic relationship of the epithelium and mesenchyme during salivary gland initiation: the role of *Fgf10*

**DOI:** 10.1242/bio.20135306

**Published:** 2013-08-09

**Authors:** Kirsty L. Wells, Marcia Gaete, Eva Matalova, Danny Deutsch, David Rice, Abigail S. Tucker

**Affiliations:** 1 Department of Craniofacial Development and Stem Cell Biology, King's College London, Floor 27, Guy's Tower, London Bridge, London SE1 9RT, UK; 2 Faculty of Medicine, Pontificia Universidad Católica de Chile, Alameda 340, Santiago, 8331150, Chile; 3 Institute of Animal Physiology and Genetics, v.v.i., Academy of Sciences of the Czech Republic, 602 00, Brno, Czech Republic; 4 Department of Physiology, University of Veterinary and Pharmaceutical Sciences, 612 42 Brno, Czech Republic; 5 Dental Research Laboratory, Institute of Dental Sciences, Hebrew University, Hadassah, Faculty of Dental Medicine, Jerusalem 91120, Israel; 6 Department of Orthodontics, Institute of Dentistry, University of Helsinki, Helsinki 00014, Finland; 7 Oral and Maxillofacial Diseases, Helsinki University Central Hospital, Helsinki 00290, Finland

**Keywords:** Salivary gland, *Fgf10*, Epithelial–mesenchymal interactions, Recombination, GFP

## Abstract

Salivary glands provide an excellent model for the study of epithelial–mesenchymal interactions. We have looked at the interactions involved in the early initiation and development of murine salivary glands using classic recombination experiments and knockout mice. We show that salivary gland epithelium, at thickening and initial bud stages, is able to direct salivary gland development in non-gland pharyngeal arch mesenchyme at early stages. The early salivary gland epithelium is therefore able to induce gland development in non-gland tissue. This ability later shifts to the mesenchyme, with non-gland epithelium, such as from the limb bud, able to form a branching gland when combined with pseudoglandular stage gland mesenchyme. This shift appears to involve Fgf signalling, with signals from the epithelium inducing *Fgf10* in the mesenchyme. *Fgf10* then signals back to the epithelium to direct gland down-growth and bud development. These experiments highlight the importance of epithelial–mesenchymal signalling in gland initiation, controlling where, when and how many salivary glands form.

## Introduction

Salivary gland development is a dynamic process involving epithelial–mesenchymal interactions. Salivary glands develop in a series of well-characterised stages, pre-bud (epithelial thickening), initial bud, pseudoglandular, and canalicular (Tucker, 2007). The major salivary glands, submandibular, sublingual and parotid, develop from neural crest derived mesenchyme and ectoderm derived epithelium (Jaskoll et al., 2002; Rothova et al., 2012). Information about the genes important during these different stages is starting to be accumulated, and this sits on a wealth of information produced from classic tissue recombination experiments. Although a lot of information regarding branching morphogenesis is starting to be accumulated, initiation of the glands is less well understood.

When E13 salivary gland epithelium was combined with other sources of non-glandular mesenchyme, such as from the maxilla, the epithelium failed to branch and formed a cyst (Grobstein, 1953b). When combined with other branching mesenchyme, such as metanephric mesenchyme, the SG epithelium formed coiled tubules rather than its usual branches (Grobstein, 1953a; Grobstein, 1953b). In contrast when salivary gland mesenchyme at E13 and E14 was combined with a host of other developing epithelium (mammary, early pancreatic, nasal, palatal or oral epithelium) a branching gland developed with the morphology of a salivary gland (Kusakabe et al., 1985; Kratochwil, 1969; Tucker, 2007; Tyler and Koch, 1977). It was therefore suggested that salivary gland epithelium depended completely on its organ specific mesenchyme for correct morphogenesis (Kratochwil, 1969).

The early recombination experiments used salivary gland epithelium and mesenchyme at stages after the overt appearance of the glands. In development timing is often a crucial factor. This was clearly shown in recombination experiments using the tooth as a model. In the tooth it is the epithelium that has the initial instructive information and can generate teeth when combined with neural crest derived mesenchyme (Lumsden, 1988; Mina and Kollar, 1987; Tucker et al., 1999b). Thus E10.5 oral epithelium combined with trunk neural crest, or second branchial arch, can induce the formation of a tooth. At the early bud stage (E12.5), however, the instructive signals pass to the mesenchyme. At this stage onwards it is the tooth mesenchyme that has the ability to induce tooth formation when combined with many different non-oral epithelia (Mina and Kollar, 1987). In keeping with this the tooth epithelium is no longer capable of inducing tooth development when combined with non-dental mesenchyme. As the early formation of the tooth and salivary glands share many characteristics, it is possible that a similar transfer of instructive information may also be occurring in the salivary glands. To investigate this we performed recombinations of salivary gland epithelium with non-salivary gland mandible mesenchyme and salivary gland mesenchyme with non-salivary gland epithelium, as has previously been reported, but performed these recombinations at earlier stages of development.

Fgf signalling has been shown to be a key player in formation of early glands, with *Fgf10* expressed in the mesenchyme around developing salivary glands, lacrimal glands and lung buds, while its receptor (*Fgfr2b*) is expressed in the overlying epithelium (Nitta et al., 2009; Makarenkova et al., 2000; Bellusci et al., 1997; Steinberg et al., 2005; Jaskoll et al., 2002). Importantly loss of *Fgf10* or its receptor in the mouse leads to aplasia of lungs, lacrimal and salivary glands, indicating the central importance of this signalling pathway for initiation of these branching organs (Ohuchi et al., 2000; Govindarajan et al., 2000; De Moerlooze et al., 2000). In human patients mutations in *FGF10*, or *FGFR2b*, lead to LADD (Lacrimal auriculo dento digital) syndrome and ALSG (aplasia of lacrimal and salivary gland) syndrome (Entesarian et al., 2005; Milunsky et al., 2006; Rohmann et al., 2006). In the mouse, a slight invagination of the salivary gland epithelium is observed at E12.5 in the *Fgf10* and *Fgfr2b* nulls, indicating that this first sign of a gland can proceed in the absence of *Fgf10* signalling (Jaskoll et al., 2005). The epithelium, however, fails to invaginate further to form a bud. In lacrimal glands addition of *Fgf10* has been shown to lead to the formation of ectopic glands, thus *Fgf10* is not only necessary for formation of lacrimal glands but is sufficient for their formation (Makarenkova et al., 2000; Govindarajan et al., 2000). The role of *Fgf10* signaling was therefore further investigated in our recombinations and in *Fgf10* null mice. For our experiments we concentrated on the development of the submandibular gland (SMG) as it forms in a clear position under the tongue and develops well in culture.

## Materials and Methods

### Mice

Mice were set up for matings at approximately midnight or midday. Embryos were obtained at E (embryonic day) 10.5 to E15.5. To aid accurate staging, morphological landmarks, such as development of tongue and eye were used to confirm the age of the embryo before recombination. GFP (Green Fluorescent Protein) reporter mice were used to visualise the growing glands as they developed and to ensure that the separation of epithelium and mesenchyme was free from contamination with the other tissue. *Fgf10* mutants were generated as previously described (Min et al., 1998; De Moerlooze et al., 2000; Rice et al., 2004). All experiments were performed according to home office guidelines using schedule 1 approved culling methods.

### Recombinations

The mandible, second pharyngeal (branchial) arch and limbs were dissected from embryos at embryonic day (E) 10.5, E11.5, E12.0 and E12.5. Dissected tissue was placed in dispase (made up to 2units/ml in calcium and magnesium free PBS and filtered). Dispase acts by removing basement membrane to cleanly separate mesenchymal and epithelial tissue. Tissue was left for 10–20 minutes at 37°C, by which time the epithelium started to peel away from the mesenchyme. The reaction was stopped by placing the tissue in medium (D-MEM/F12 plus penicillin/streptomycin and 1% Glutamax (Invitrogen)) and the two tissues completely separated using tungsten needles. Care was taken to remove the invaginating salivary gland epithelium from the cultures without breaking the tissue or leaving epithelial cells behind. Mesenchyme explants were cultured on transparent nucleopore filters (VWR) supported on metal grids on the surface of the medium. Epithelium was then draped over the mesenchyme. In whole mandible epithelium recombinations, the orientation of the epithelium was determined by the thickening in the incisor and salivary gland regions that could be clearly observed in the isolated epithelium. The recombinations were then covered with a thin layer of matrigel (BD Bioscience), a gel of basement membrane that solidifies at 37°C. Matrigel plays an important role in aiding culture of salivary gland epithelium but is unable to induce branching of isolated SG epithelium (Takahashi and Nogawa, 1991; Steinberg et al., 2005). Explants were cultured at 37°C/5% CO_2_ up to 9 days in D-MEM/F12 plus penicillin/streptomycin and 1% Glutamax (Invitrogen), changing the medium every 2 days, and were photographed every day.

### Fgf10 rescue experiments

Whole presumptive submandibular glands (E12.5 and E13.5) were dissected out of the lower jaw and cultured on filters as above. Embryonic tails were used for genotyping although Fgf10 homozygous mutants were easily distinguishable due to their lack of limbs. Heparin beads were soaked overnight at 4°C in Fgf10 protein (R&D Systems) at 100 µg/ml. Beads were washed briefly in PBS before use and implanted into the salivary gland capsule. Control beads were soaked in an equivalent concentration of BSA. Pairs of submandibular glands from the same mouse were cultured together, one implanted with an Fgf10 bead, the other with a control bead. Alternatively Fgf10 protein was added directly into the medium at 2 µg/ml.

### Slice cultures

E11.5 mouse mandibles of CD1 embryos were dissected out and sliced using a McIlwain tissue chopper (Mickle Laboratory Engineering Co., Ltd. UK) into frontal slices 200 µm thick (Rothová et al., 2011). Slices showing a distinct epithelial thickening/invagination at the base of the tongue were then selected for further processing. Slices were cultured as above with an Fgf10 bead placed on one side and a BSA control bead on the other side. To distinguish the beads affigel blue beads were used for the controls and white heparin beads for the Fgf10.

### Fgf8 beads

Heparin beads were incubated with 100 µg/ml Fgf8 protein (R&D Systems) overnight in the fridge. Protein loaded beads and control BSA beads were added to mandibles that had had their epithelium removed using dispase (see above). Mandibles were then cultured for 48 hours and fixed.

### GFP immunohistochemistry

Recombinations were fixed in 4% PFA and taken through a methanol series and embedded in wax via isopropanol and tetrahydronapthalene. Anti-GFP antibody (Abcam, #ab290) was used at a concentration of 1:500 on paraffin sections, followed by a biotinylated anti-rabbit secondary antibody (Dako) at a concentration of 1:200. The antibody was detected using an Elite kit (Vector) and the GFP was visualised using a DAB reaction (Vector). Slides were counterstained in eosin.

### In situ hybridization

Cultures were fixed in 4% PFA overnight and dehydrated through a methanol series. DIG wholemounts in situ were performed following a modified Wilkinson protocol (Wilkinson, 1995). Radioactive 35S in situ hybridization were performed as previously described (Tucker et al., 1999a). Mouse *Fgf10* plasmid was a kind gift from Ivor Mason.

### Confocal

GFP recombinations were mounted on a slide, bordered by two layers of sellotape to provide a shallow well into which Prolong® Gold anti-fade reagent with DAPI was added (Invitrogen, #P36935). The tissue was slightly squashed with a coverslip and imaged using a confocal microscope Leica SP5. Images were processed using Adobe Photoshop*®*. When needed, smart sharpen filter, hue-levels and despeckle plug-in was applied.

### BrdU

*Fgf10* pregnant heterozygous females were injected intraperitoneally with 20 mg of BrdU per 1 kg weight of the injected animal. The mice were sacrificed 2 hours after injection and the E15.5 embryos were collected. The embryonic heads were fixed in 4% paraformaldehyde (PFA) overnight and embedded in paraffin. The paraffin sections were de-paraffinized, re-hydrated, boiled in 10 mM sodium citrate pH 6 for 5 mins 4×, blocked with 0.2% gelatine and 10% lamb serum for 2 hours and incubated at 4°C overnight with primary antibody to BrdU (diluted 1:100 in block solution). BrdU positive cells were visualised using a DAB reaction (Vector).

## Results

### Salivary gland mesenchyme can direct development of a branching gland in non-gland epithelium

**Fig. 1.**
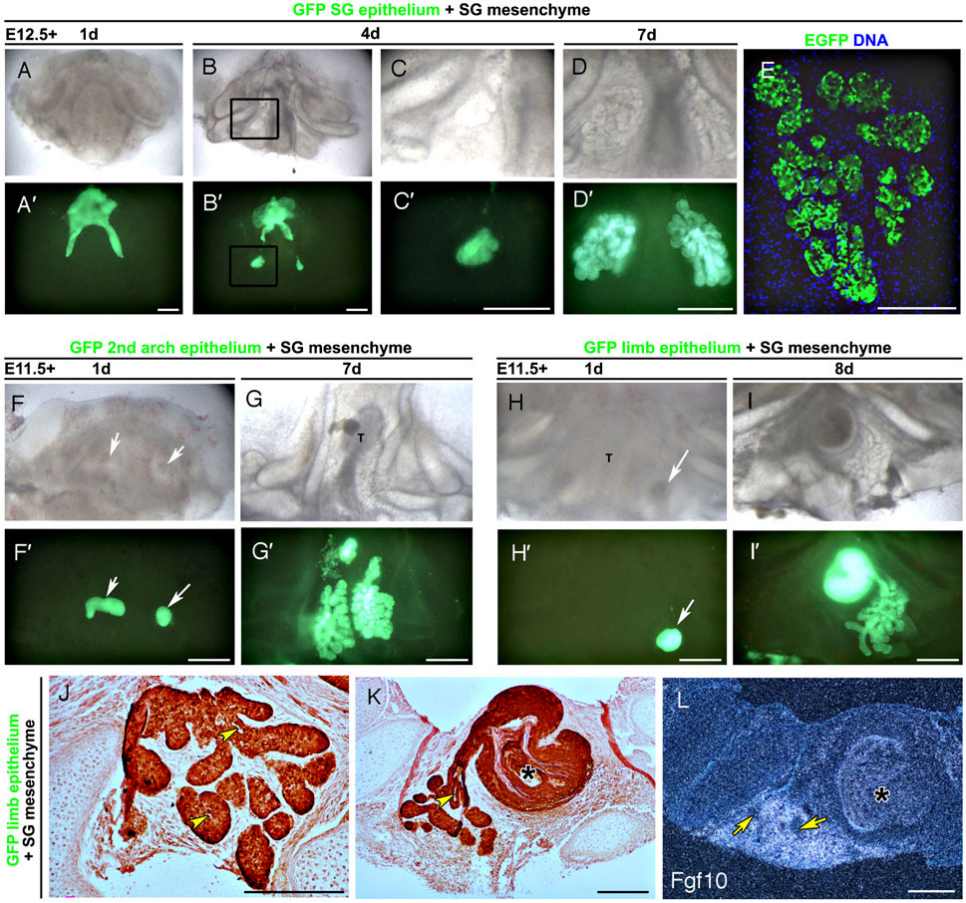
Salivary gland mesenchyme can drive gland development in non-gland epithelium. (**A–D**) E12.5 GFP salivary gland epithelium combined with E12.5 wildtype mesenchyme from a dissected mandible. (A–D) Bright field. (**A′–D′**) Dark field highlighting GFP expressing epithelium. (A,A′) Day 1. (B,B′) Day 4. (C,C′) High powered view of box in panel B. The epithelium has started to branch over the salivary gland mesenchyme. (D,D′) Day 7. The gland continues to branch as a normal salivary gland. Epithelium in non-gland regions is lost. (**E**) Confocal image showing GFP is restricted to the branching epithelium of the gland. (**F**,**G**) E12.5 GFP 2^nd^ pharyngeal arch epithelium combined with E11.5 salivary gland mesenchyme from a dissected mandible. (F,**F′**) Day 1. Grafted epithelium arrowed. (G,**G′**) Day 7. The epithelium has formed a branching gland on either side of the tongue (T). (**H–L**) E11.5 GFP limb epithelium combined with E12.5 salivary gland mesenchyme from a dissected mandible. (H,H′) Day 1. Grafted epithelium on one side of the tongue (T) arrowed. (I,I′) Day 8. A branching gland forms over the presumptive salivary gland mesenchyme. (J,K) GFP immunohistochemistry on sections. The branching epithelium is derived from the GFP donor and forms lumens (yellow arrows), budding off from a cyst-like structure of keratinised tissue (asterix in panel K). (L) In situ hybridisation for *Fgf10*. The branching gland (yellow arrows) is found within the Fgf10 positive region. The cyst (asterix) is found within *Fgf10* negative tissue. Scale bars: 500 µm (A–D,F–I), 250 µm (E,J–L).

The SMGs are evident as thickenings of the oral epithelium at E11.5 and proceed to an elongated bud shape (known as the initial bud) by E12.5 (Tucker, 2007). Their early development thus mirrors the stages of tooth development where a thickening is viewed at E11.5, leading to an early bud at E12.5. As E12.5 is the critical stage when the information for tooth development transfers from the epithelium to the mesenchyme we concentrated our salivary gland recombinations around this time point. In order to visualise the salivary gland as it develops we combined mandible mesenchyme from a wildtype mouse with mandible epithelium from a GFP (Green fluorescent protein)-reporter mouse (or vice versa). The developing salivary gland could then be followed over the culture period. When E12.5 GFP salivary gland epithelium was combined with wildtype salivary gland mesenchyme a small bud was visible after 2 days of culture, which started to branching after 4 days in culture, with a well-developed multi-lobed gland forming after 7 days (Fig. 1A–D,A′–D′; Table 1) (*n* = 6). Confocal imaging confirmed that the epithelium of the gland derived solely from the GFP labelled epithelium (Fig. 1E). To test the instructive nature of the mesenchyme, presumptive salivary gland mesenchyme from E11.0, E11.5 and E12.5 was covered with non-salivary gland mandibular, or second pharyngeal arch epithelium from a GFP donor. Second pharyngeal arch tissue does not form any branching structures during normal development in the mouse (Grevellec and Tucker, 2010). In each case a clear branching SG-like structure developed (Fig. 1F,G,F′,G′; Table 1). To further confirm the instructive potential of the salivary gland mesenchyme, E11.5 and E12.5 salivary gland mesenchyme was covered with GFP labelled limb bud epithelium. A salivary gland again formed in each case where the limb epithelium was placed directly over the salivary gland mesenchyme (*n* = 3/5) (Fig. 1H,I,H′,I′; Table 1). Immunohistochemistry for GFP showed that the limb epithelium had transformed to a branching structure (Fig. 1J,K). Interestingly branching structures were only observed where the epithelium was overlying a region of *Fgf10* expression (*n* = 3) (Fig. 1L). Limb epithelium placed outside the Fgf10 expression zone failed to branch and formed a cyst (as seen in Fig. 1K). Interestingly, a longer delay in gland initiation was observed in cultures with limb epithelium compared with mandibular epithelium, indicating reprogramming of the epithelial tissue. A range of epithelium is therefore able to respond to a signal from the presumptive salivary gland mesenchyme and form a salivary gland.

**Table 1.**
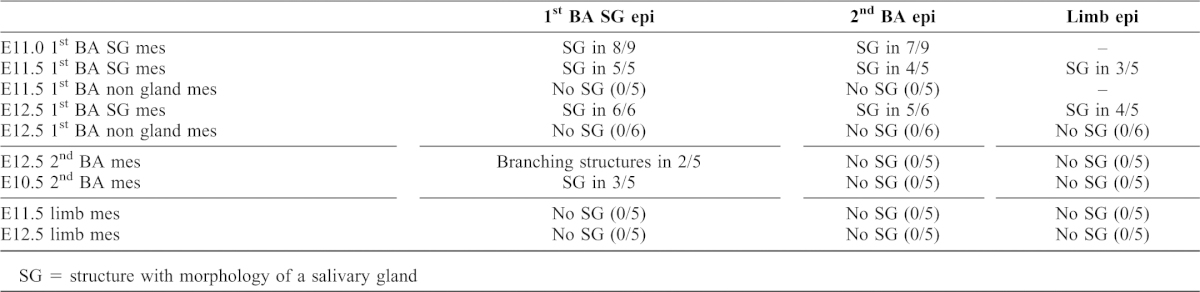
Recombinations and outcomes.

### Early salivary gland epithelium can direct development of a gland in non-gland mesenchyme

**Fig. 2.**
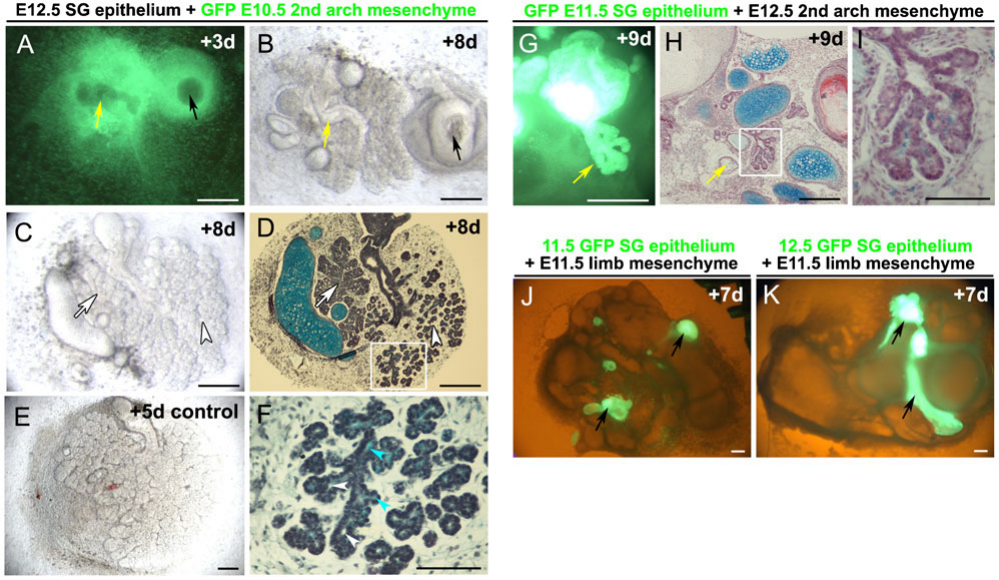
Early gland epithelium drives salivary gland formation. (**A–D**,**F**) E12.5 salivary gland epithelium combined with GFP labelled E10.5 2^nd^ pharyngeal arch mesenchyme. (A) LHS Salivary gland epithelium (yellow arrow). RHS 2^nd^ pharyngeal arch epithelium (black arrow) combined with GFP mesenchyme. Day 3. Dark field GFP. The non salivary gland epithelium remains as a rounded sphere while the salivary gland epithelium has started to elongate and a cleft has formed at the end of a bud-like structure. (B) Same culture after 8 days. The salivary gland epithelium has formed a classic branching structure with a central cavitated duct (yellow arrow), while the non-gland epithelium has formed a cyst (black arrow). (C) Salivary gland epithelium on second arch mesenchyme, cultured for 8 days. (D) Histology section of panel C. Two gland types are observed, mimicking the normal arrangement of the submandibular (arrowhead) and sublingual gland (arrow). (**E**) Control unrecombined salivary gland cultured from E12.5 for 5 days. (F) Magnification of boxed area in panel D, showing presence of developing lumens (white arrowheads) and alcian blue stained polysaccharides (blue arrows). (**G–I**) E11.5 GFP salivary gland epithelium combined with E12.5 2^nd^ pharyngeal arch mesenchyme. (G) The epithelium attempts to make some extended branched structures after 9 days in culture (yellow arrow). (H) Histology section. Some branching structures with partially developed ductal lumens are evident after 9 days in culture. (I) Magnification of boxed area in panel H, showing alcian blue stained polysaccharides in the lumens. (**J**) E11.5 GFP salivary gland epithelium (arrows) combined with E11.5 limb mesenchyme after 7 days in culture. (**K**) E12.5 GFP salivary gland epithelium (arrows) combined with E11.5 limb mesenchyme after 7 days in culture. In both cases no branching structures form. Images smart sharpened in photoshop. Scales bars: 250 µm (A–E,G,H,J,K), 100 µm (I,F).

To test the hypothesis that early salivary gland epithelium might be able to induce formation of a gland in non-gland mesenchyme, salivary gland epithelium from E11.0 to E12.5 embryos was combined with second pharyngeal (branchial) arch mesenchyme from a GFP reporter mouse. At these early stages the salivary gland epithelium can be distinguished after dissociation from the mesenchyme as thickenings or elongated buds. At the same time second arch or limb epithelium was applied to second arch mesenchyme and cultured as a control. After 3 days in culture the salivary gland epithelium started elongating, while the non-gland epithelium remained as a rounded ball (Fig. 2A). Recombination of salivary gland epithelium on early second arch mesenchyme (E10.5 and E11.0) resulted in a clear gland-like structure after 8 days, while the non-gland epithelium formed a cyst (Fig. 2B; Table 1). In some cases it appeared that a distinct submandibular and sublingual gland formed, mimicking the side-by-side arrangement of these glands *in vivo* (Fig. 2C,D). After 8 days in culture the glands appeared similar in shape to control untreated salivary glands cultured from the same time point for 5 days, although the glands were smaller (compare Fig. 2C with Fig. 2E) (*n* = 3/5). These recombined glands formed acini and duct structures with central lumen and stained for alcian blue, indicating the production of polysaccharides (Fig. 2D,F). The ability of a gland to develop in second arch mesenchyme, however, was influenced by stage. When the experiments were repeated with older second arch mesenchyme (E11.5 and E12.5) some parts of the epithelium started to undergo branching morphogenesis (Fig. 2G) (*n* = 2/5) but the extent of branching was limited and the resultant glands were much smaller (compare Fig. 2D with Fig. 2H). Lumen formation and the presence of polysaccharides in the forming ducts, however, were still present (Fig. 2I). Thus non-gland mesenchyme appears to lose its competence to form a gland as it develops.

To challenge the presumptive salivary gland epithelium further, E11.5 and E12.5 GFP labelled salivary gland epithelium was recombined with limb mesenchyme from the same stage. In this case the limb mesenchyme, was unable to respond to the signal from the salivary gland epithelium and no branching occurred, even after 13 days in culture (Fig. 2J,K; Table 1). Early salivary gland epithelium can therefore drive development of a salivary gland, but only in competent mesenchymal tissue. From E13.0 onwards, once branching of the gland was underway, the salivary gland epithelium was no longer able to induce gland formation when combined with non-gland mesenchyme, agreeing with previous recombination experiments (data not shown).

### Induction of *Fgf10* by early SG epithelium

**Fig. 3.**
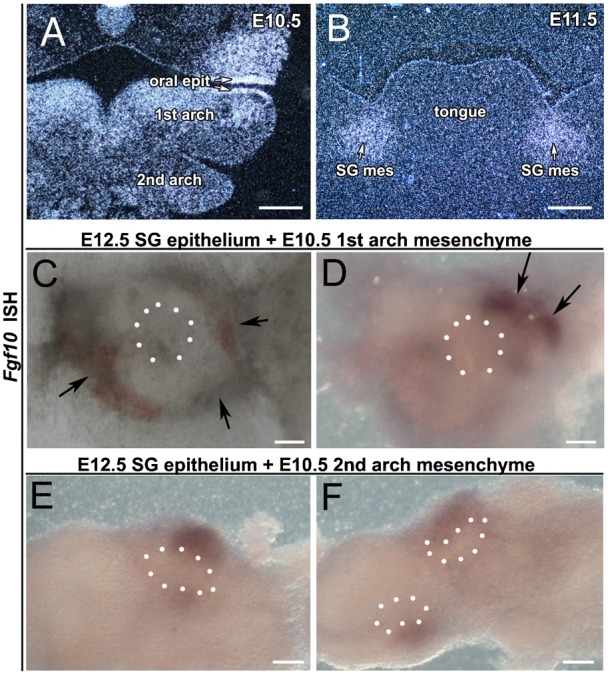
Induction of Fgf10 by early salivary gland epithelium. (**A**,**B**) Expression of *Fgf10* by radioactive in situ on frontal sections. (A) At E10.5 *Fgf10* is expressed both in the oral epithelium and widely in the first arch mesenchyme. (B) By E11.5 expression is restricted to two areas on either side of the developing tongue underlying the first signs of thickening of the salivary gland epithelium. (**C**,**D**) E12.5 salivary gland epithelium on E10.5 first arch mesenchyme (epithelium removed). (C) A halo appears around the epithelium after 2 days in culture. (D–**F**) Wholemount *Fgf10* DIG in situ on cultures. (D) Induction of *Fgf10* in first arch mesenchyme on one side of the recombination. (E,F) E12.5 salivary gland epithelium on E10.5 second pharyngeal arch mesenchyme (epithelium removed). *Fgf10* is induced asymmetrically in the mesenchyme. Epithelium outlined by white dots. Scale bars: 250 µm (A,B,E,F), 100 µm (C,D).

**Fig. 4.**
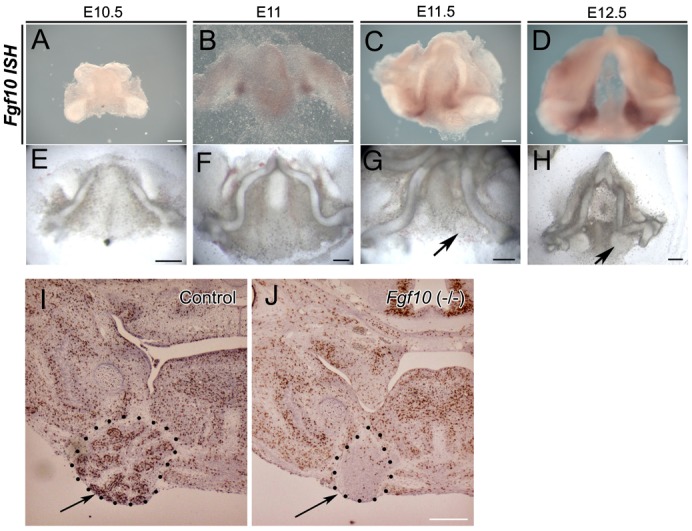
Condensation of the salivary gland mesenchyme can occur in the absence of the epithelium or *Fgf10* signalling. (**A–H**) Mandibles with epithelium removed. (A–D) *Fgf10* wholemount DIG in situ hybridisation after two days in culture. (E–H) Morphology after 4 days in culture. (A,E) E10.5 mandible. (B,F) E11.0 mandible. (C,G) E11.5 mandible. (D,H) E12.5 mandible. After E11.0, Fgf10 expression is maintained and the mesenchyme starts to condense into a capsule after removal of the epithelium (arrows). (I,J) BrdU showing proliferating cells (brown) in (**I**) WT and (**J**) Fgf10 mutant littermate. The epithelium in the wildtype is highly proliferative, but a capsule still forms in the mutant (arrow). Images smart sharpened in photoshop. Scale bars: 250 µm.

At E10.5 *Fgf10* is widely expressed in the mesenchyme and epithelium of the first and second pharyngeal arch (Fig. 3A). Expression in the mesenchyme at this stage is dependent on the presence of the overlying epithelium (Fig. 4A). By E11.5, however, expression is lost in the epithelium and downregulated in the mesenchyme. In the first arch expression becomes concentrated in two mesenchymal patches under the tongue, at the sites of the future salivary glands (Fig. 3B). We therefore tested whether the early salivary gland epithelium could induce *Fgf10* expression in pharyngeal arch mesenchyme (where the epithelium had been removed). Salivary gland epithelium at E11.5 and E12.5 produced a halo of condensed mesenchyme around the epithelium (Fig. 3C) and was able to induce *Fgf10* expression in the underlying mesenchyme of E10.5 first pharyngeal arch (Fig. 3D) and second pharyngeal arch mesenchyme (Fig. 3E,F) after 2 days in culture. No induction of *Fgf10* was observed in limb mesenchyme, or in areas of second arch mesenchyme where epithelium was not placed (data not shown). Interestingly expression of *Fgf10* was not induced symmetrically around the grafted epithelium, indicating that some parts of the mesenchyme were more competent to respond then others, or that the inducing signal from the epithelium was released asymmetrically.

These experiments indicate that an early signal from the SG epithelium induces expression of *Fgf10* in the mesenchyme during normal salivary gland development. To look at this further we took mandibles and removed the epithelium at a series of stages from E10.5 to E12.5. The mandibles were then cultured without the epithelium for 2 days. During this time the cultures continued to grow, despite absence of the epithelium, as indicated by the development of Meckel's cartilage. After culture the expression of *Fgf10* was assessed. In the early mandible cultures no expression of *Fgf10* was observed (Fig. 4A), however in those cultures where the epithelium had been removed at E11.0 onwards *Fgf10* expression was observed in two patches on either side of the tongue in the presumptive salivary gland mesenchyme, indicating that by E11.0 *Fgf10* expression in the mesenchyme is independent of signals from the epithelium (Fig. 4B–D) (*n* = 5 for each stage). Interestingly, when the mandibles were cultured for longer the presumptive *Fgf10* expressing SG mesenchyme started to condense and form a capsule despite the absence of any epithelium (Fig. 4E–H) (*n* = 5 for each stage). Thus, once the initial induction signal has occurred, the salivary gland mesenchyme can continue to develop and form a condensed mesenchymal capsule. In keeping with this observation, the salivary gland mesenchyme forms a capsule in Fgf10 knockout mice, despite the fact that the epithelium arrests at the thickening stage (Fig. 4I,J). The condensation of the mesenchyme is therefore independent of the presence of Fgf10. The mesenchymal capsule in WT and mutant mice showed low proliferation, in contrast to the high proliferation of the salivary gland epithelium in WT embryos, indicating that the capsule is formed by condensation of cells rather than increased proliferation (Fig. 4I,J).

### Fgf10 directs down-growth of the salivary gland epithelium

**Fig. 5.**
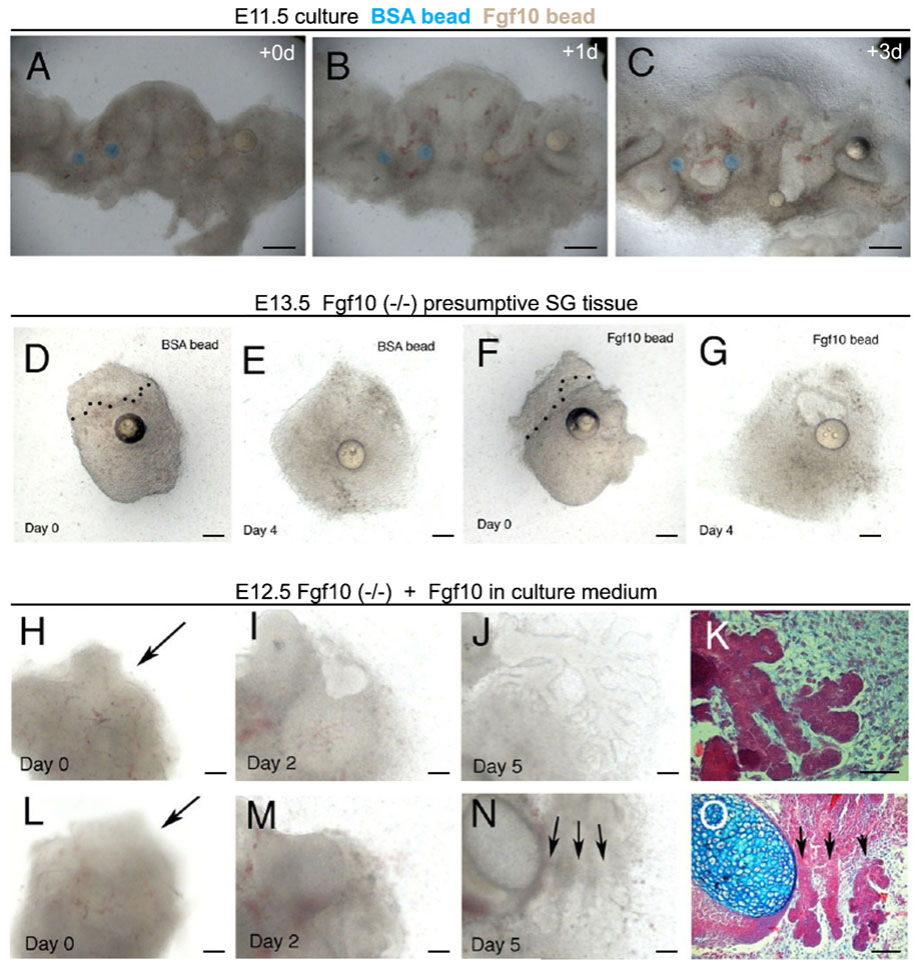
Fgf10 leads to elongation of the salivary gland. (**A–C**) Slice cultures of the developing mandible at E11.5. Frontal live section 200 µm. Tongue in the middle with developing salivary glands on either side. (A) Day 0. BSA control beads on LHS (blue). Fgf10 loaded Beads RHS (white). Submandibular glands at the initial bud stage. (B) Day 1. The epithelium on the RHS has turned towards the nearest Fgf10 bead, while the control side grows straight down. (C) Day 3. The epithelium on the RHS has elongated past the normal gland position pushing the bead downwards. Rescue of gland development. (**D–O**) Fgf10 mutant presumptive salivary gland tissue. (D,F) E13.0 gland with beads added. Day 0. Epithelium outlined by dotted lines. (E) Day 4. No growth of the epithelium in after addition of a PBS control bead. (G) Day 4. Growth of the epithelium towards the Fgf10 bead, and the start of branching morphogenesis. (H–O) E12.5 gland with Fgf10 added to the medium. (H,L) Day 0. Arrows point to gland epithelium arrested at the thickening stage. (I,M) Day 2. In panel I a clear bud can be observed extending into the mesenchyme that has condensed into a capsule. (J,N) Day 5. Branching glands are in evidence. In panel N three distinct glands have formed (arrows). (K,O) Histological sections of the cultures showing branching and the onset of lumen formation. Image shows merged image from two adjacent sections to show the whole gland. Scale bars: 250 µm (A–C), 100 µm (D–O).

These experiments demonstrate that the early SG epithelium first signals to the mesenchyme inducing formation of an *Fgf10* positive SG capsule. It would then be predicted that the *Fgf10* expressing mesenchyme would signal back to the epithelium to induce down-growth of the gland epithelium. To test this, beads soaked in Fgf10 protein were placed close to the developing submandibular gland at the initial bud stage. In order to visualise the developing glands during culture we used live slices through the mandible and placed Fgf10 beads (white heparin beads) on one side of the tongue and BSA control beads (affigel blue beads) on the opposite side. The localised source of Fgf10 led to a downwards growth of the salivary gland epithelium, with the epithelium extending in the direction of the nearest Fgf10 bead (Fig. 5A,B). After three days in culture the epithelium had overshot the normal position for gland development and pushed the bead far away from its original insertion site. In contrast the control beads remained at the same relative position within the culture (Fig. 5C) (*n* = 6).

### Rescue and induction of ectopic salivary glands in Fgf10 mutants

In *Fgf10* mutants the loss of Fgf signalling leads to a failure of the epithelium to invaginate into the mesenchymal capsule (Fig. 4I,J). As the capsule forms in *Fgf10* mutant mice, the presumptive salivary gland mesenchyme and overlying epithelium can be dissected out and cultured. At E13.0, when the submandibular gland has started to branch in wildtype mice, no extension of the epithelium was observed in *Fgf10* nulls (Fig. 5D,F). Over several days in culture with BSA control beads no change in the epithelium was observed (Fig. 5E). When Fgf10 beads were added, however, the rudimentary epithelium started to grow down towards the bead and began to branch (Fig. 5G) (*n* = 3/5). The epithelium is thus still able to respond in these mutants when given the correct signal. When Fgf10 was provided in the medium at E12.5 large branching glandular structures formed (Fig. 5H–K), however, in some cases multiple glands were initiated (Fig. 5L–O) (*n* = 3/4). Application of exogenous Fgf10 can therefore leads to ectopic formation of salivary glands.

### Fgf8 does not induce *Fgf10* in the mesenchyme

**Fig. 6.**
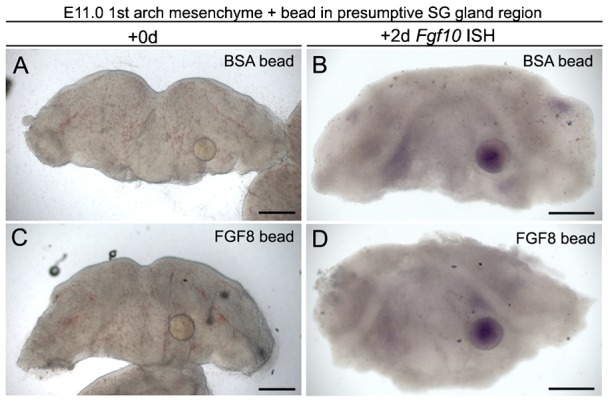
Fgf8 does not induce *Fgf10* in the gland mesenchyme. (**A**,**C**) E11.0 1^st^ BA mesenchyme minus epithelium plus heparin bead inserted in presumptive salivary gland region at the base of the forming tongue. Day 0. (A,**B**) BSA control beads. (C,**D**) Fgf8 beads. (B,D) In situ hybridisation for *Fgf10* after 48 hours in culture. Weak expression is observed in the presumptive salivary gland mesenchyme on either side of the tongue and in the future molar placodes but there is no upregulation around the beads. The beads take up some staining solution and turn purple. Scale bars: 250 µm.

The previous results show the crucial role of *Fgf10* in signaling from the mesenchyme to the epithelium to direct gland development. The identity of the signal from the epithelium that starts off this cascade of epithelial–mesenchymal signaling is unclear. From the recombination and epithelium removal experiments this signal appears to occur between E10.5 and E11.0, and leads to a localisation of *Fgf10* expression to the future sites of gland development. The signal also leads to condensation of the underlying mesenchyme to form a capsule, independent of *Fgf10* expression. A possible candidate for this signal is *Fgf8*, as *Fgf8* has been shown to regulate *Fgf10* expression in the developing limb (Moon and Capecchi, 2000; Xu et al., 1998) and *Fgf8* is expressed in the oral epithelium from E10.0 (Tucker et al., 1999b). To test this Fgf8 loaded beads were added to cultures of first pharyngeal arch mesenchyme where the epithelium had been removed at E11.0. The addition of Fgf8 led to a translucent zone developing around the bead after 2 days in culture (data not shown), as has previously been documented (Kettunen and Thesleff, 1998). Weak expression of *Fgf10* was observed in the mesenchyme at the position of the future glands and teeth in both Fgf8 and BSA control bead experiments, however, no upregulation of *Fgf10* by Fgf8 was observed (Fig. 6) (*n* = 8). Therefore Fgf8, signalling from the epithelium, appears unlikely to induce expression of *Fgf10* in the underlying mesenchyme.

## Discussion

A transfer of information from the epithelium to the mesenchyme was discovered in the developing salivary gland, which mirrored the changing roles of the epithelium and mesenchyme previously shown to occur during tooth development. In submandibular salivary glands, instructive information was found to reside in the oral epithelium at stages before E12.5. After this age the epithelium was no longer instructive and did not induce gland development in non-gland tissues. The mesenchyme, however, was competent to induce a gland in non-gland tissue from E11.5, indicating that for a period both tissues have the instructive capacity to induce a gland. This time point coincides with the localisation of *Fgf10* expression to the presumptive gland mesenchyme. If the epithelium is removed prior to E11.0, expression of *Fgf10* is lost, while, after this time point, expression of *Fgf10* is retained in the presumptive gland mesenchyme.

*Fgf10* plays a central role in the development of the salivary glands, directing down-growth of the epithelium into the condensed mesenchymal capsule. This agrees with previous work that has shown that addition of Fgf10 to isolated gland epithelium at E13.0 stimulates proliferation specifically at the tip of the developing ducts, leading to elongation of the ducts (Steinberg et al., 2005). Fgf10 has also been shown to be a positive signal for duct growth in the lacrimal glands (Tsau et al., 2011).

Like teeth and SGs, hair follicle development involves epithelial–mesenchymal interactions, and the epithelium (epidermis) and mesenchyme (dermis) are thought to play changing roles depending on the developmental stage (Hardy, 1992). Classical tissue recombination experiments suggested that the condensing dermis carries the initial signal, inducing hair placode formation in the overlying epidermis (Dhouailly, 1973). However, more recent work has shown the existence of epidermal to dermal communication from the earliest stages of hair placode patterning, as well as the very early patterned expression of epidermal hair placode markers in the absence of dermal condensation (Fliniaux et al., 2008; Mou et al., 2006; Huh et al., 2013). These findings suggest a transfer of instructive information from epidermis to dermis at a very early stage of hair follicle induction. *Fgf10* is expressed in the dermis during hair placode development (Petiot et al., 2003). Mice deficient in *Fgfr2-IIIb*, produce fewer and developmentally retarded hair follicles (Petiot et al., 2003), while *Fgf10*-null mice exhibit fewer whiskers with disorganized structure (Ohuchi et al., 2003). Therefore it is tempting to speculate that the teeth, SGs and hair follicles all share a similar transfer of instructive information at their earliest developmental stages, and that for salivary glands and hair follicles, at least, this involves Fgf10.

Findings from other papers have suggested that Fgf10 can be regulated by Fgf8 signalling from the epithelium (Moon and Capecchi, 2000; Xu et al., 1998). In support of a role for *Fgf8*, salivary gland development is disrupted in conditional *Fgf8* mutant mice, where *Fgf8* is lost in the oral epithelium (Jaskoll et al., 2004). However, as with the *Fgf10* mice, an initial thickening does develop in these mice indicating that *Fgf8* is not necessary for controlling the induction of the gland at this site (Jaskoll et al., 2004). Fgf8 protein has been found localised to both the gland epithelium and mesenchyme at early stages of SMG development, and intriguingly the level of Fgf10 protein in the aborted glands was suggested to be reduced in conditional *Fgf8* mutants (Jaskoll et al., 2002; Jaskoll et al., 2004). Despite this we find no evidence that Fgf8 can induce *Fgf10* in pharyngeal arch mesenchyme. This agrees with previous culture studies where beads soaked in Fgf8 protein were able to induce *Fgf3* but not *Fgf10* in dental mesenchyme at E11 and E12 (Kettunen et al., 2000). In these experiments, although *Fgf10* expression was shown to be dependent on the epithelium, *Fgf10* was not induced by Fgf4, Bmp2, Shh, Tgfβ1 or Wnt6 (Kettunen et al., 2000). The epithelial signal that induces *Fgf10* in the salivary glands mesenchyme therefore remains unknown.

In our recombinations, second arch mesenchyme was capable of forming a gland if given the correct signals (i.e. early gland epithelium) while limb mesenchyme was not. This is similar to the finding that teeth only developed in neural crest derived mesenchyme (Lumsden, 1988). This indicates that there is something innately different about these two sources of mesenchyme. Such constraints on where tissues have the potential to form may have evolved to limit the distribution of organs, for example, restricting the development of salivary glands to the oral regions of the animal. In some birds, such as pigeons, large mucous glands form from the second arch, reminiscent of salivary glands, and therefore the second arch in mammals may have retained a competence to form this tissue type (reviewed by Grevellec and Tucker, 2010).

In *Fgf10* mutants, loss of *Fgf10* signalling results in a failure of the epithelium to progress past the initiation stage but the signal that comes from the epithelium does induce condensation of the mesenchyme into a capsule in the region of the presumptive gland. Addition of Fgf10 was able to rescue the down-growth of the epithelium, even at E13.0, over a day after normal gland extension would have started, indicating the epithelium overlying the gland still retains the potential to grow and branch if given the correct signal. The localised expression of *Fgf10* under the epithelial thickening at E11.5 appears likely to control not only the direction of growth but also how many glands develop, as addition of Fgf10 in culture medium led to the formation of additional glands. This appears similar to the lacrimal glands where ectopic glands can be induced by addition of ectopic Fgf10 (Makarenkova et al., 2000).

The transfer of instructive information from one tissue to another makes the possibility of forming a complete salivary gland from non-gland tissue a possibility. In theory early salivary epithelium could be recombined with a competent mesenchyme, and then removed after the instructive signal has passed to the mesenchyme. Non-gland epithelium from a range of sources could then be applied to the originally non-gland mesenchyme to create a gland. Understanding tissue interactions and their timing is therefore an important step in being able to create a bioengineered gland.
