# Dynamic relationship of the epithelium and mesenchyme during salivary gland initiation: the role of *Fgf10*

**DOI:** 10.1242/bio.20149084

**Published:** 2014-06-23

**Authors:** Kirsty L. Wells, Marcia Gaete, Eva Matalova, Danny Deutsch, David Rice, Abigail S. Tucker

This Correction relates to *Biol. Open* 2013, 10:981–989.

Unfortunately, there were errors in the first published version of this article. These errors are detailed below and the original article has been changed accordingly.

The statements regarding funding were incomplete and the following text has now been included in the Acknowledgements section:

D.R. is supported by the Academy of Finland, The Sigrid Jusélius Foundation and Helsinki University Central Hospital Research Foundation.

We apologise for any confusion caused.

